# Rabies*Read before the Section on Internal Medicine of the Fifth-Sixth District Medical Society, at Corpus Christi, Texas, September 8, 1914.

**Published:** 1914-10

**Authors:** R. H. L. Bibb


For Texas Medical Journal.

### Rabies.*

BY R. H. L. BIBB, M. D.

I do not write on the subject of Rabies because it is alarmingly frequent, nor because it is invariably fatal, but mainly because the disease, and its management, is so poorly understood by the profession at large that the simple preventive measures against it, which have been shown to be so efficacious, wherever and whenever they have been properly applied, that these measures often miscarry when most needed.

### DEFINITION.

Rabies is defined as an acute infectious disease of certain of the lower animals, communicable to man by the salivary secretions of such of them as may be afflicted with it; characterized by a variable incubation period; by running an extremely short course; localization of the virus in the central nervous system and in the salivary glands; the invariable presence of the *neuroryctes hydrophobiae* in the cytoplasm of certain brain cells, and by protection through rabic vaccine.

### HISTORY.

Some three hundred years before Christ, Aristotle wrote of rabies among dogs, who, by biting them, communicated it to all other animals except man. Celsus, in a compilation, a hundred years after Christ, wrote of rabies as it appeared in man, so that it would seem fair to infer that, like the poor, rabies is a disease which we have always had with us.

With the exception of Australia, where all imported dogs are subjected to six months quarantine, and where all dogs are required to constantly wear muzzles, and where no case of rabies in either man or beast has ever been known to occur, hydrophobia has been found co-extensive with the habitation of man.

By muzzling and by quarantine, more especially the former, England, where rabies was once alarmingly frequent, has been entirely free of it for a number of years.

Rabies attacks all the mammalia, but is seen most frequently in the dog, the wolf, the cat, horse, goat, cattle, sheep and hog. The deer and fox seem somewhat immune. It is claimed that the rabbit helps to propagate the disease in nature, as do rats and mice, and that the skunk—the "hydrophobia skunk"—even when

*Read before the Section on Internal Medicine of the Fifth-Sixth District Medical Society, at Corpus Christi, Texas, September 8, 1914.

not afflicted, is a natural carrier of rabies, although there is no proof that these contentions are based on facts.

Birds enjoy a partial, if not complete immunity; and old pigeons and old fowls frequently recover from what is thought to be rabies.

Neither age nor sex are factors in susceptibility; but children are more often bitten than adults, because more in contact with dogs in their sports; in dogs rabies is more frequent in those of from one to four years of age. Neither does season affect the incidence of rabies, and where the opposite would seem to be true, it is accidental.

According to Babes, bites about the eyes, nose and lips by rabid wolves are fatal in 100 per cent of such bites, and that mortality from bites by cats in the same localities is 70 per cent, and 60 per cent for dog bites. In other parts of the face bites from wolves are fatal in 80 per cent; in dogs and cats it is 50 per cent. In deep bites on other uncovered parts of the body it is 40 per cent for the wolf and cat and 30 per cent for the dog. In single and deep bites on the neck or finger, it is 20 per cent for the wolf and the cat, and 15 per cent for the dog. In deep bites, on well covered parts of the body, mortality is 15 per cent for the wolf, 10 per cent for the cat, and 3 per cent for the dog. In superficial bites, on uncovered portions of the body, the mortality is 10 per cent for each of the three animals.

The mortality among dogs bitten by rabid dogs is about 40 per cent. Bites from the herbivora and from man are very slightly, if at all, dangerous; and there is no record, no known case of the transference of rabies from the bite of man to man.

Only a comparatively small percentage of humans and animals bitten by rabid animals develop rabies. The number in all cases is closely proportionate to the depth, the extent, and the site of the bite, and to the animal biting. Whilst this holds true, in the main, it must not be forgotten that it is very probable that the specific germ of rabies present in a given case may vary, in numbers and in virulence, from hour to hour, and that this fact may explain the variable number of persons developing the disease after being bitten, who had no special treatment.

According to Stimson—(Pub. Health Repts., 1912)—of 4625 persons bitten by 3393 rabid dogs in 1911, and treated in the various Pasteur institutes in the United States, 98 died. This is certainly a highly gratifying mortality showing; but, gratifying as it is, it is questionable if it contains the actual number bitten, or anywhere near the mortality during the time specified.

PATHOLOGY.

It is not known how the *neurorycles hyprophobiae* act, immediately after entering the body. They have been traced, however, from the site of the bite along the nerve fibers to the brain. Occasionally, as an accidental and a very transient occurrence, they have been found in the blood, where, supposedly, they are quickly destroyed by the phagocytes. Once within the cytoplasm of the nerve cell—never in the nucleus—they gradually develop until the cell is destroyed.

Many histological changes, not diagnostic, are found in the brain cells and in the nerve trunks, such as pin-point hemorrhages, areas of softening, groups of small spheroidal cells surrounding many of the orterioles and the larger nerve cells, especially marked in the anterior and posterior horns, and named by Babes "rabic tubercles," and which he once thought pathognomonic of rabies; but their absence in so many cases of rabies render their absence or presence of no diagnostic importance, more especially now that the Negri bodies, discovered and announced by Negri in 1903, which are recognized to be specific for rabies, are of such easy demonstration.

These bodies—sometimes called the Negri bodies—and sometimes the *"neurorycles hydrophobiae,"* are said to belong to the protozoa family, to be spore forming and to be capable of extruding pseudopods, are minute particles of protoplasm from one to forty microns in diameter, and are easily found as inclusions in the cytoplasm of the nerve cells of Amon's horn, and in the Perhinje cells of the cerebellum. They are round, oval, oblong or triangular in shape—their shape, seemingly, depends upon whether they are impigned upon or not by the surrounding tissues,—and in stained specimens are seen to contain minute pigmented granules, in varying numbers, from one to seven or eight, interspersed throughout the body of the parasite. These bodies may be found throughout the cephalic mass and in the spinal cord, but are rarely, if ever, absent from the localities indicated above, and are easily demonstrated therein.

ETIOLOGY.

Rabies is conveyed to man exclusively by bites from animals afflicted with the disease—chiefly by dogs; hence it is that in countries where dogs are permitted to roam around unmuzzled, most cases of it are seen.

### INCUBATION.

The incubation period of rabies in man is usually from six to eight weeks. It may, however, be as short as two weeks and as long as three months.

### SYMPTOMS.

The premonitory symptoms of rabies as observed in human beings present nothing indicative of the terrible disease that follows them, and last rarely longer than a day or two. They consist of general restlessness, anxiety, depression, insomnia and frequently with pain or soreness at the bitten point, and not infrequently of darting pains simulating neuralgic pains from any cause.

In the spasmodic stage there is great excitement and restlessness with frequent severe and very painful spasms of the larynx and pharynx, which are excited by an attempt to swallow, or by the mere sight or thought of water or other fluid, or by any external stimulus such as a sound or touch or by a draft of air striking any portion of the body. The muscles of respiration are involved, the voice becomes hoarse with a characteristic quality, somewhat like a hoarse bark. The patient's mental condition is intact. Sometimes there are maniacal outbursts that require restraint. The temperature ranges from between 38-39. The pulse is rapid and grows progressively weaker. These symptoms last about forty-eight hours—one should be able to make a diagnosis by this time—and are succeeded by the:

Terminal or paralytic symptoms in which the muscles relax, the jaws hang apart, saliva flows; the face becomes smooth and expressionless, coma supervenes; breathing becomes irregular; the pulse frequent and intermittent, and finally stops as a merciful death draws its sable pall over the harrowing scene in from two to eighteen hours.

Usually the temperature increases just before death, and has been observed to reach as high as 44.5 (112 F.). Sugar and acetone, but no albumen, are found in the urine.

### DURATION.

Counting from the first appearance of premonitory symptoms all rabid patients die within eight days.

### DIAGNOSIS.

It is hardly possible for hydrophobia to be confounded with any other disease after the convulsive symptoms appear, unless it be with lyssophobia, a curious hysterical condition which occasionally develops in hysterical subjects who have been bitten by some animal known to be carriers of rabies. All of the nervous phenomena of true rabies may be present; but there is no rise of temperature, and as the symptoms rapidly disappear, under proper treatment, the physician need not long remain in doubt. Delirium tremens, tetanus and the action of several of the poisonous drugs may be easily excluded.

It is reasonably certain that cases of "dumb" or "paralytic" rabies, escape detection in both man and beast, especially in the latter. It is claimed that 20 per cent of rabies in dogs is of this variety. The stage of excitation is short or absent, paralysis being the first symptom noted. The lower jaw hangs open, causing the "drop-jaw," which suggests the dog may have a bone lodged in its throat. The animal is incapable of biting, although its saliva may be as virulent as that of a biting animal. The entire body of animals with "dumb rabies" becomes quickly paralyzed and the animal soon dies.

On account of the occasional occurrence of paralysis in cases treated with antirabic vaccine—103 cases in 211,774 treated and reported up to 1912—this form of rabies has acquired new interest, and the question is asked if some, if not all of these may not have been cases of "dumb rabies" instead of poisonings from the antirabic treatment. But, inasmuch as the same group of symptoms, with deaths, have occurred in both man and animals who were not bitten but subjected to treatment, the inevitable conclusion is: that the treatment itself does, occasionally, cause both paralysis and death.

TREATMENT.

Inasmuch as the curative treatment of rabies is absolutely valueless and therefore futile, prophylaxis becomes of prime importance; and, thanks to Pasteur's brilliant discovery, it is as certain, when intelligently employed, as any proposition that ever confronted the medical fraternity; and it is in aiding and urging proper prophylaxic methods the profession can exert its most valuable influence in ridding humanity of the dangers of rabies.

As being the most important, the easiest, the most simple and the least costly, I would place the *muzzling of all dogs, and the prompt killing of all dogs, whether in the owner's enclosure or the commons, found unmuzzled.* This is certainly an easy and a simple process, and that it is efficient, one need only remember that by it England exterminated hydrophobia within her home borders in a few years, and that by it, Australia has succeeded in keeping it without her confines up to this good hour, which is quite enough to say in its favor.

The next most important method of prophylaxis is the "specific" or "antirabic"—Pasteur's priceless gift to humanity in 1885—which, as employed today, is efficient in more than 99 per cent of cases; but it is believed if the muzzling of dogs was strictly enforced, as it should be, the specific treatment would be rarely, if ever, necessary.

The time at my disposal is too limited to permit a detailed account of the various steps in the "specific" treatment of rabies. It is sufficient to state that all wounds, however superficial or insignificant, inflicted by animals capable of conveying hydrophobia, should be treated as infected wounds. Free bleeding should be encouraged, and the injury cleaned as quickly as possible with any fluid antiseptic at hand, and whenever possible, well cauterized with fuming nitric acid. Babes recommends bathing the wounds with antirabic serum, but I suspect it would be valueless.

The next important question—and it is a momentous one—is: should the patient be subjected to specific treatment?

If we could determine in every case the presence or not of rabies, our duty would be clear cut and easy; but, unfortunately, several factors prevent this ready solution of the problem. The biting animal may have been a stray and disappeared after the biting; he may appear perfectly healthy; he may present indefinite symptoms; he may be killed before clinical or microscopical manifestations appear, or his brain may have been sent to a laboratory improperly and insecurely packed and may have arrived in too bad a condition for microscopic examination—the latter is the case in a large number of brains sent to the Pasteur Institute at Austin.

If these factors cannot be ruled out, rabies cannot, and unless rabies can be ruled out, positively, we should be extremely guarded, indeed, we *should never,* under the circumstances, advise against treatment, especially in a community where rabies has been recently reported.

Antirabic treatment should be advised, therefore, whenever and wherever the following conditions obtain:

When the animal shows clinical or microscopical signs of rabies.

When an animal disappears just after biting and cannot be found, even though he be a "stray."

When the biting animal has been killed before it can show microscopic evidence of rabies.

When the animal's brain reaches the laboratory in a condition that makes a microscopic examination impossible.

When an animal has been killed and no evidence can be had for or against rabies where the disease is prevalent.

When fresh cuts or abrasions have been exposed to the saliva of rabid animals. The slightest fresh cut or abrasion may be more dangerous than deeper clean cut bites that bleed freely; and it is probable that most, if not all, cases cited where rabies is said to have developed without bite or contact, are really contact cases.

In addition to the advice that should govern us in advising for or against specific treatment, I wish to add:

When an apparently healthy animal bites a person, that animal should be tied up or housed for at least two weeks, and if at the expiration of that time it shows no symptoms of rabies, specific treatment is unnecessary. Of course, should the animal show earlier symptoms, treatment should be begun at once, and the patient would have lost nothing by the delay.

Wherever it becomes necessary to send an animal's brain to a laboratory for examination, one of the cheapest, and perhaps one of the best is to simply cut the animal's head off, place it in an ordinary water bucket, fill the bucket, so as to cover the head, with cracked ice, and cover the bucket with a strong gunny bag, such as is used for shipping grain.

The variable incubation period of rabies produced by infecting rabbits with virus obtained from the brains of dogs who died of rabies—"street virus," induced Pasteur to try the effect of infecting these animals, in succession, from one to another. By this method he produced a virus that was not only fixed in its incubation period, but greatly intensified in its virulence as well. This is the virus used in the present day specific treatment, and is known as "fixed virus."

When a rabbit dies from rabies induced by inoculation with fixed virus, the cord is removed asepticly, stripped of its membranes, cut across in its middle and the two ends suspended by cords in bottles containing a quantity of caustic potash, and placed in a room which is kept constantly at 20 degrees C., for desiccation by which its virulence becomes attenuated. After remaining thus for twenty-four hours it is called "one day cord." After forty-eight hours it is known as "two day cord," and so on.

Specific treatment is begun, at present, by giving an initial dose of ½ cm. of eight day cord rubbed up in 2½ c.c. of normal saline solution, hypodermically, in some part of the abdominal wall, gradually decreasing the age of the cord until one day cord is used and twenty-one injections given.

The scheme of the Hygienic Laboratory in Washington is as follows: Initial dose of eight day cord then of 4, 5, 3, 3, 2, 2, 1, 5, 4, 4, 3, 3, 2, 2, 4, 3, 2, 1. This is known as the "intensive treatment" and is the only one now employed at that institution in adult cases. It is somewhat modified for children. In Berlin the treatment is yet more intensive. There the initial dose, for adults and children is 2 c.c. of one part of *three day cord* to five parts of sterile physiologic salt solution, then 2, 1, 1, 3, 2, 1, 1, 3, 2, 1, 1, 3, 2, 1, 1, 3, 2, 1, 1, 1. In 1909-1910 this method, in 819 cases was attempted with a mortality of .6 of 1 per cent.

The immunity following the specific treatment, which takes place in about fifteen days after the last inoculation, is thought to be variable. In one patient, fourteen months after treatment, a second infection was followed by fatal rabies. In dogs it has been found complete eighteen months after treatment.

There are numerous other methods of applying the specific treatment, but inasmuch as none of them have met with much favor, it is deemed best not to waste time by discussing them here.

Prophylactic treatment may be carried out at the patient's home, but with such an efficient institution as is the Pasteur Institute at Austin, it is much safer to send your patients there, and I know that Dr. Wilhite, the accomplished physician in charge, will do all for them that can be done anywhere on earth.

All that I have said to you about rabies is the common property of the profession; and you can find it in any of the recent textbooks on medicine.

